# Animal Abuse as an Indicator of Domestic Violence: One Health, One Welfare Approach

**DOI:** 10.3390/ani12080977

**Published:** 2022-04-10

**Authors:** Daniel Mota-Rojas, Stefany Monsalve, Karina Lezama-García, Patricia Mora-Medina, Adriana Domínguez-Oliva, Ramiro Ramírez-Necoechea, Rita de Cassia Maria Garcia

**Affiliations:** 1 Neurophysiology of Pain, Behavior and Assessment of Welfare in Domestic Animals, DPAA, Universidad Autónoma Metropolitana (UAM), Mexico City 04960, Mexico; 2192801631@alumnos.xoc.uam.mx (K.L.-G.); 2212801915@alumnos.xoc.uam.mx (A.D.-O.); neconsultorio@yahoo.com.mx (R.R.-N.); 2 Facultad de Ciencias Agrarias, Programa de Especialización en Bienestar Animal y Etología, Fundación Universitaria Agraria de Colombia, Calle 170 No 54 A 10, Bogotá 111321, Colombia; smonsalveb@unal.edu.co; 3 Facultad de Medicina Veterinaria y Zootecnia, Fundación Universitaria San Martín, Carrera 18 No 80 45, Bogotá 110221, Colombia; 4 Animal Welfare and Behavior Center, Facultad de Estudios Superiores Cuautitlán, Universidad Nacional Autónoma de México (UNAM), Mexico City 54714, Mexico; morapat@cuautitlan.unam.mx; 5 Veterinary Medicine of the Collective and Veterinary Social Work, Legal Veterinary Medicine, Federal University of Paraná, Curitiba 80060-000, Brazil

**Keywords:** animal cruelty, animal welfare, violence, the link, one welfare, veterinary social work

## Abstract

**Simple Summary:**

Animal abuse is widely recognized as both a risk factor for and a potential consequence of interpersonal violence. In children, especially, factors such as dysfunctional families, antisocial personality, physical, psychological, or intimate abuse, and frequent exposure to domestic aggression or animal abuse have been confirmed as factors that can predispose young people to perform acts of animal cruelty. It is important to recognize warning signs such as those identified as the McDonald triad (bedwetting, pyromania, animal cruelty). A one health, one welfare approach, incorporating physicians, veterinarians, other health care professionals, social workers, and humane education, is critical for the recognition, management, and prevention of domestic violence, involving both humans and other animals.

**Abstract:**

For years now, the importance of animal cruelty has been gaining recognition in the industrialized cities of the West. Animal cruelty encompasses any act that causes a non-human animal unnecessary pain or suffering, including negligence, abandonment, abuse, torture, bestiality, and even theriocide. This represents a red flag for society as a whole because people who commit such acts can escalate violence and direct it to other individuals. Animal cruelty and interpersonal violence—as well as other socially undesirable conduct such as bullying, antisocial personality disorder, rape, and serial murder—are closely related, so timely diagnoses of either one can help prevent acts of aggression. It is necessary, therefore, to analyze and try to understand whether there are early indicators that may help identify potentially violent individuals. It is well known that kids from homes with actual violence in their homes show a high tendency to reproduce such behaviors with both animals and other people. In conclusion, much research and rethinking of the importance of the veterinarian in detecting animal abuse and cruelty is needed to help detect and prevent cases of interpersonal violence that may arise over time.

## 1. Introduction

Animal cruelty, maltreatment, and abuse began to emerge as socially important issues in industrialized cities of the West in the 18th century [1,2], and include a wide variety of harmful behavior towards animals, from unintentional neglect to malicious killing [3]. The definition of these terms can vary among countries and research/review papers, or sometimes are used as synonyms, causing confusion [3,4]. For this review, the terms cruelty, abuse, and maltreatment are used as synonyms because studies linking interpersonal violence to animal abuse use the terms interchangeably and refer to non-acceptable social behaviors that intentionally cause pain, suffering, distress, or even the death of an animal [5], including acts of non-accidental injury, abandonment, neglect, or torture that may occur as kicking, hitting, stabbing animals, failing to provide food and water [6], veterinary services [7], organized fights, sexual abuse (bestiality), burns, wounds with projectiles or sharp weapons, shootings, suffocation, poisoning [8], or even theriocide [9,10]. Animal cruelty can affect any animal species but the ones most often victimized are dogs and cats [11,12,13].

Many studies have shown a significant relationship between animal abuse with interpersonal violence, including domestic or family violence (violent acts between family members and intimate partners that occur at home but not exclusively) [14], and community violence (violence between unrelated individuals that may or may not know each other, it tends to occur outside the home [7,14,15,16,17,18,19,20]. One reason for this association is that in some cases of domestic violence abusers harm animals to intimidate and impose psychological control over their victims [18,21,22]. Similarly, acts of animal cruelty in children have been reported. The socioemotional well-being of children who witness domestic violence—often perpetrated by the mother’s intimate partner—may be compromised, leaving them at a higher risk of committing animal abuse themselves [23]. Thus, animal abuse can serve as an indicator of child abuse, family violence, or violent criminal behavior [24]. The first report to show a relationship between interpersonal violence and animal abuse was published by Pinel in 1806. It documented the behavior of a man who was violent towards both animals and humans [25].

MacDonald [15] observed a relationship between, on the one hand, a triad of enuresis, pyromania (fire-setting), and animal cruelty during childhood and, on the other, a predisposition of those children to become violent criminals as adults. Mead [26] reported that cruelty perpetrated upon animals by children could indicate the development of a behavioral disorder. Animal cruelty in children is associated with factors such as lack of empathy, poor socioemotional development, or child abuse [27]. There are macroenvironmental factors such as economic stress, a high unemployment rate, and high crime rates that can lead to parental stress and, therefore, domestic violence, but that are also associated with an increased risk of child animal abuse by not understanding that the animals are sentient beings [28].

Other authors have found that intimate partners who are violent towards women also often commit acts of abuse against pets [7,17,29,30,31]. In many cases, abusers perpetrate acts of abuse against companion animals to intimidate and exert psychological control over their victims [18,22,32]. The incidence of cases of animal cruelty is alarming. A study by Vaughn et al. [33] found that 2% of 43,000 adults interviewed declared having participated in an act of animal cruelty at some point in their lives.

Today, we have tests that help measure levels of cruelty, empathy, and insensitivity, such as the Animal Cruelty Inventory (ACI) [34], the Griffith Empathy Measurement (GEM) [35], and the Inventory of Callous-Unemotional Traits (ICU) [36]. However, much work remains to be carried out to promote the well-being of both humans and animals that have been, or still are, victims of abuse or mistreatment, and to develop and implement programs that serve to educate people, treat and correct abusers, and, in the specific case of animal abuse, demand greater participation and responsibility on the part of veterinarians in identifying acts of cruelty. Veterinarians are key because their everyday professional practice brings them into contact with animals that have been victims of non-accidental trauma, neglect, or sexual abuse [37,38,39,40,41,42]. In the same way, the forensic veterinary approach can help to determine the risk the offender represents for other animals and for society [13,43]. Unfortunately, and despite the fact that many schools of veterinary medicine in developed countries have improved their curricula to include courses on animal welfare, a deficiency in training in animal abuse, interpersonal violence, and veterinary forensics in veterinary colleges has been reported [38,39,40,41]. The level of training in animal abuse and the role that veterinarians play in cases of animal abuse are still largely ignored in many countries, especially in Latin America [24].

The goals of this review are to analyze the literature on the relationship between animal cruelty and interpersonal violence, to describe the current status of animal welfare legislation, and to suggest a one health, one welfare approach, to prevent animal cruelty or acts of violence of any kind. 

## 2. Material and Methods

**Figure 1:**
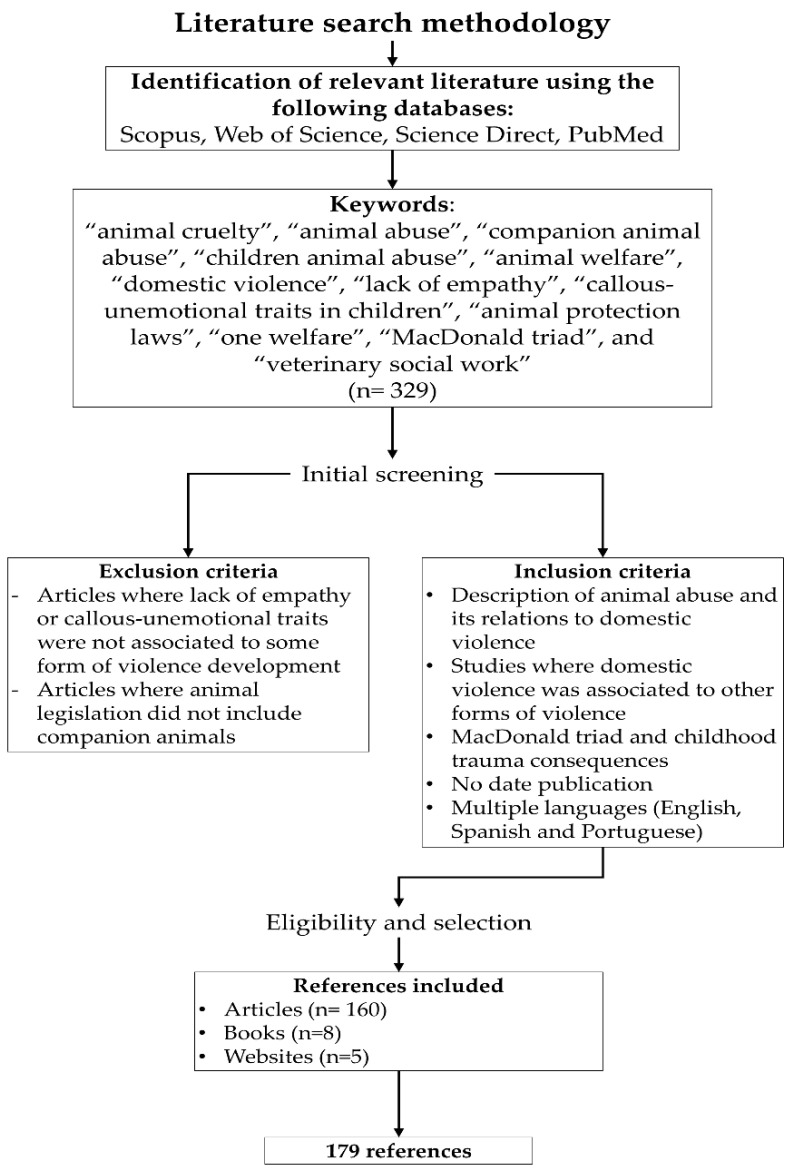
Search methodology.

We searched for relevant articles using PubMed, Scopus, Science Direct, and Web of Science. Identification of relevant articles was performed using the following keywords: “animal cruelty”, “animal abuse”, “companion animal abuse”, “children animal abuse”, “animal welfare”, “domestic violence”, “lack of empathy”, “callous-unemotional traits in children”, “animal protection laws”, “one welfare”, “MacDonald triad”, and “veterinary social work”. Included references were articles related to the description of animal abuse and how it is related to domestic violence, the risk factors for the development of animal cruelty in children, and its association to callous-unemotional traits or warning signs that can be used to opportunely detect and prevent domestic violence or other crimes. Likewise, articles about the legislation to protect companion animals, and one health approach programs were also included. There was no settled date of publication to select the articles. The search was conducted in multiple languages (English, Spanish, and Portuguese). The search methodology and the selection of the 179 references used for this review are described in Figure 1.

## 3. Animal Cruelty as an Indicator of Domestic Violence

The connection between domestic violence and animal cruelty has been demonstrated in diverse studies [42,43,44,45]. Generally, violent criminals have histories of animal abuse at some stage in their lives more often than non-violent criminals [5]. DeGue and DiLillo [46] suggest that identifying cruelty against pets is a red flag for domestic violence or violence against children.

The World Health Organization [47] describes violence as “the deliberate use of physical force or power, whether threatening or effective, against oneself, another person, or a group or community, that causes or has many probabilities to cause injury, death, psychological damage, developmental disorders or deprivation”. Derived from this, Mora-Medina et al. [48] define the term intrafamily violence as “abuse by a perpetrator towards a victim within the family nucleus”. Abuse of animals is more commonly reported in children who have been exposed to domestic violence [8]. Similarly, young people who are involved in acts of animal cruelty tend to have an increased risk of perpetrating violent acts against their sentimental partners, loved ones, or older family members [49]. An analysis by Bright et al. [8], of interviews of 81,000 juvenile aggressors, found that 466 (0.6% of the total sample) had participated in acts of animal cruelty. It highlighted that 37.6% of the respondents who reported having perpetrated animal abuse committed their first offense before age 12. This finding is similar to those of previous studies, which mention that 76–88% of the homes where animal cruelty occurs also presented cases of abuse against children [26].

**Figure 2:**
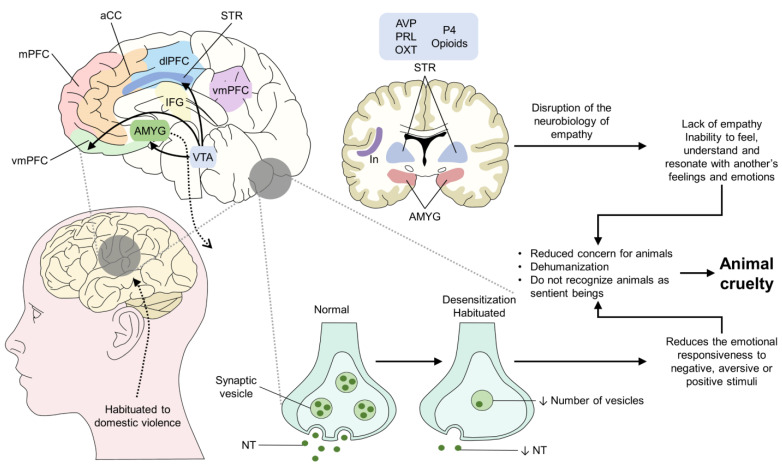
Association among lack of empathy, habituation to domestic violence, and animal cruelty. aCC: anterior cingulate cortex; AMYG: amygdala; AVP: vasopressin; dlPFC: dorsolateral prefrontal cortex; GLU: glutamate; IFG: inferior frontal gyrus; In: insula; mPFC: medial prefrontal cortex; NT: neurotransmitter; OXT: oxytocin; P4: progesterone; PRL: prolactin; STR: striatum; TPJ: temporoparietal junction; vmPFC: ventromedial prefrontal cortex; VTA: ventral tegmental area.

Although the neurobiology of empathy and the effect of violence is not well understood to date, Figure 2 tries to represent the structural and neurobehavioral changes that could explain the development of animal cruelty and its association with domestic violence and lack of empathy. According to the literature, when a person, particularly a child, constantly witnesses domestic violence, apart from the behavioral problems that it entails, children can present difficulty developing empathy or a lack of empathy towards others [50]. When referring to a lack of empathy, the neurological pathway that involves the brainstem, cerebral cortex, and the limbic system with its representative structures—mPFC, aCC, vmPFC, AMYG, IFG, dlPFC, vmPFC, VTA, STR, and In—participates in developing a person’s emotional state and her/his understanding of those of others, a trait that is modulated by individual differences and person-environment aspects [51,52]. When an individual shows traits such as a lack of empathy, this not only represents a social issue but also involves a potential disruption of the neurobiological pathways of empathy development [53]. Additionally, it is said that children and adolescents who are exposed to violence can become emotionally desensitized or habituated to violence [54]. During a process of habituation, some structural changes may cause alterations in the neurotransmitter levels by a reduction in the number of vesicles in the presynaptic neurons and a low concentration in the postsynaptic cleft [55]. The main neurotransmitters implicated in empathy deficits in children are oxytocin and serotonin [52], and dysregulation of endogenous opioids and oxytocin has also been suggested as a potential cause for a dysregulated empathic network [56]. This dysregulation could decrease the transmission of, and responsiveness to, stimuli, whether negative, aversive, or positive [55,57]. As a result of a compromised empathy development, children may be unable to understand the emotions of others or deny that animals are sentient beings [58,59], and these could be possible factors that predispose them to commit acts of animal cruelty.

Companion animals turn into victims of family abuse when the abuser tries to cause fear, intimidation, vengeance, and control over the female partner and children [60]. Thus, companion animal abuse may be an effective method of coercive control used against women who are very close to their pets [61]. Even concern for the welfare of a companion animal can affect a victim’s decision making about whether to stay with or leave a violent partner [62]. In fact, many women who have been victims of abuse refuse to go to shelters because they cannot take their pets, but must leave them at home where they may be at the mercy of abusers [20] who subject them to cruelty to emotionally harm their victims due to the close bond they share with their pets [63]. Witnessing violence towards, or the death of, a companion animal, can be traumatic in children highly attached to their pets, causing anxious/depressed symptoms [64,65]. Surveys conducted by Carlisle-Frank et al. [66] in New York revealed that 53% of domestic violence victims reported having witnessed companion animal abuse. That percentage is similar to that observed by Allen et al. [29] in Ireland (57%), while Ascione et al.’s [16] analysis carried out in the state of Utah found that 54% of the victims of abuse interviewed had witnessed violent acts, including malicious killing, involving their pets, and that 67% of the children interviewed while living in shelters reported having seen animal abuse. Table 1 provides a comparison of five regions.

In Australia, Volant et al. [2] interviewed 102 women from various social strata, divided into two groups: those who had experienced domestic violence, and those who had not. All those subjects had companion animals. Of the females in the domestic violence group, 52.9% reported having seen animal abuse by their partners, including kicking, hanging, hitting the animals with objects, breaking their necks, or shooting them with a firearm. In contrast, the women in the group without domestic violence reported 0% cases of domestic animal abuse. Another important observation was that 29% of the women in the domestic violence group reported that their children committed animal abuse, while in the group of women without domestic violence, animal abuse by children occurred in only 1% of cases. Therefore, over half of domestic violence cases reported worldwide include precedents of animal abuse, though Faver and Strand [21] found a lower prevalence (36%) among Hispanic women who were victims of domestic violence. Similarly, Currie [67] mentions that children who witness domestic violence have a higher predisposition to perpetuate animal abuse than children who did not grow up in this disrupted environment. Furthermore, when children or adolescents witness animal abuse, they tend to replicate this behavior in the future, both animal abuse, bullying, and juvenile delinquency [68].

Evidence of this kind shows that violence has a serious social impact inside the family nucleus and that if this environment is not respected by its members, they may find great difficulty in understanding how to live with other beings in their environment. Fortunately, there are indicators that can raise red flags to promptly detect individuals who may be prone to committing some kind of violence. These indicators suggest that violent people may potentially act aggressively towards conspecifics or companion animals, so those behaviors should be taken as warning signs in order to intervene opportunely and prevent additional acts of abuse.

## 4. Risk Factors for the Development of Animal Cruelty in Children

The prevalence of animal cruelty in children is diverse and, in many countries, still unknown. According to Campbell [69], 53% of animal abuse in companion animals is perpetrated by adults and 20% is perpetrated by children. Thus, for example, in Italy, Baldry [70] found that 50% of adolescents committed animal cruelty, while in Australia, 21% has been reported [71]. The origin of acts of animal cruelty by violent people is an urgent topic for research because it is important to identify situations that may lead a child to commit violent acts against animals. Hartman et al. [30] examined the relationship between empathy, callous-unemotional traits, and animal abuse in 290 children in the United States aged 7–12 years whose mothers had suffered intimate partner violence. They found that 16.2% had hurt an animal on at least one occasion and concluded that cruelty or insensitivity (callous-unemotional traits) and low empathy could serve as predictors of animal abuse by children. Their report is similar to one by Muñoz et al. [72], who observed that levels of empathy and cruelty—or callous-unemotional traits—predict antisocial behaviors and could lead to animal abuse by young people. These results are consistent with the findings of a meta-analysis which showed that bullying was negatively associated with cognitive and affective empathy (odds ratio OR = 0.60, OR = 0.51, respectively). Apart from the positive correlation between insensitivity and bullying (OR = 2.55) [73], the authors stated that children who lack empathy, do not understand emotional traits, and are habituated to witnessing abusive actions, will likely be unable to understand that animals are sentient beings. As a result, their social performance will be poor. Among the risk factors that predispose a child to develop animal abuse at some stage of his life, we can cite having witnessed animal abuse, being victims of physical or sexual abuse, alcoholic or drug addict parents, family dysfunction, or present some behavioral disorder [19,33,74,75,76,77]. In the studies carried out by Baldry [78], Henry and Sanders [79], and Walters [80], a positive correlation between animal abuse and subsequent bullying behavior was found. Tapia [81] reported that, in addition to bullying, aggression, fights, and robbery can also be present.

**Figure 3:**
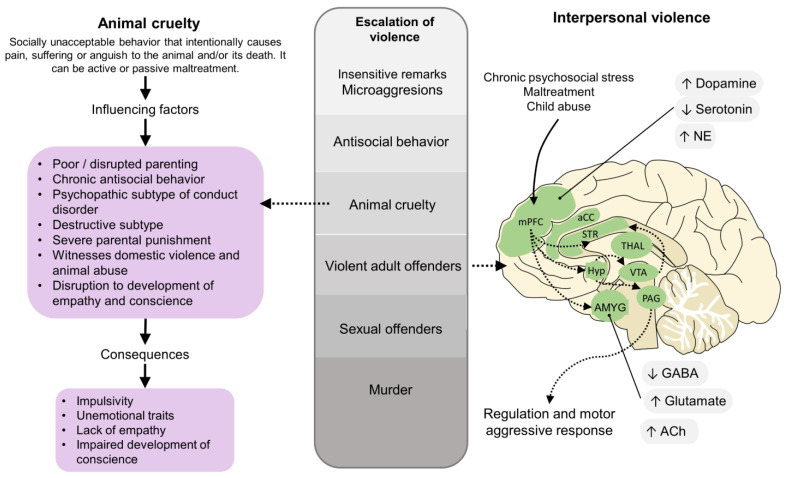
The origin of childhood animal abuse and the development of interpersonal aggression. aCC: anterior cingulate cortex; Ach: acetylcholine; AMYG: amygdala; Hyp: hypothalamus; mPFC: medial prefrontal cortex; NE: norepinephrine; PAG: periaqueductal gray; STR: striatum; THAL: thalamus.

Children engaged in animal abuse are more likely to show violent criminal behavior in adulthood, mainly when acts of animal cruelty are recurrent, engage bestiality, involve drowning, choking, burning, and the motive behind animal cruelty is fun [68,82,83,84]. Empirically, animal abuse has also been related to a tendency towards the emergence of serial killers [85]. Figure 3 schematizes the correlation that the literature has been found between a suggested escalation of violence where animal abuse and its influencing factors may cause progressive violence and aggression towards conspecifics [86]. In fact, animal cruelty has been shown to be a component of the behavioral history of psychopaths and serial killers who began their criminal lives with acts of animal abuse as children or adolescents, since callous-unemotional traits and lack of emotional reactivity are considered the precursors of psychopathy development [87]. In this sense, psychopathy is a neurological disorder characterized by a lack of remorse and empathy, impulsiveness, diminished emotional response to their surroundings, and a higher predisposition to develop criminal behaviors [88]. Adults and children with psychopathy and callous-unemotional traits lack emotional empathy but preserve the cognitive one [89]. However, it is considered that the latter represents the affective domain of the multidimensional structure of psychopathy [90]. Several factors have been shown to influence the development of animal cruelty: e.g., poor parenting, a psychopathic subtype of conduct disorder [91,92], continuously witnessing domestic violence, disruption of the development of empathy, among others [5,91]. The consequences of a lack of empathy and impaired development of conscience are associated with an escalation in violence from animal cruelty to serious offenses, such as sex crimes or murder [92,93,94]. Additionally, when an individual is exposed to chronic psychosocial stress, has a trauma history—such as abuse—or emotional dysregulation, neurotransmitters (serotonin, norepinephrine, GABA, glutamate, or acetylcholine), and cortical and subcortical regions (mPFC, aCC, STR, AMYG; THAL, Hyp, etc.) participate in the brain circuitry that regulates aggressive behavior [95]. Therefore, the promotion of motor aggressive responses towards animals or conspecifics is known to be the result of multisensory cues from the environment [96].

The risk factors that may predispose children to develop animal abuse at some stage of life include the following: having witnessed animal abuse, being victims of physical and sexual abuse, having alcoholic parents, being raised in a dysfunctional family, being exposed to domestic violence, and presenting a behavioral disorder [19,74,75,76,77]. According to Baldry’s [78] evaluation of a sample of 268 boys and 264 girls aged 9–12 from five schools, 44% reported having witnessed episodes of domestic violence. That analysis reported that those children were 3–8 times more likely to have performed acts of animal mistreatment than children who had not witnessed intrafamily violence. These results agree with Henry’s study [97] of 206 students, 141 of whom had abused animals. The latter group had witnessed animal cruelty before age 13. That study also determined a negative relationship on a scale that measured attitudes and evaluated how animals should be treated (Attitudes Toward the Treatment of Animal Scales, ATTAS) (r = −0.06, *p* < 0.001). Children who often mistreat animals are commonly abused in the community, at school, and in the family [75,78,98]. Other authors mention that there are macro-environmental factors such as disorganization, economic stress, and unemployment that can also influence the relationship between children and animals [28]. Hence, animal abuse in children is considered a form of dehumanization, where minors are not able to recognize their own pain or the suffering they inflict on others [99]. Morera et al. [100] state that, in a dehumanized society, animals are perceived as machines and not sentient beings. If the younger members of society cannot recognize animals as beings that can suffer and feel pain, a circle of violence tends to be repeated in future generations. Children who perform such acts would speak about the instability of society and its deterioration where the child does not understand the importance of living with another being. If these ideas are not corrected, they can be learned by the next generations and, therefore, reflect the advancement or regression of society.

Similarly, nine factors have been identified to explain why humans, especially children, may abuse animals: (1) to have control of them; (2) to get revenge on them; (3) to satisfy prejudices towards a particular species or breed; (4) to show aggression; (5) to impress others by showing them what they are capable of; (6) to amuse bystanders; (7) to retaliate against other people; (8) to avenge a personal aggression by harming someone else’s pet; and (9) to satisfy sadistic tendencies [1,101]. The associated studies stress, of course, that each case must be studied individually considering the person involved and her/his possible social-psychological motivations [91]. Other authors report that children may also perpetuate cruel actions towards animals out of simple curiosity or by imitating acts they have observed. This may reflect desensitization to violence, low empathy, or the absence of sentimental ties to the victim [102,103]. In the worst cases, children living in a home where intrafamily violence occurs may even kill a pet to save it from suffering torture in the future [18]. The trend observed to date shows that the main animals that children abuse or mistreat are small animals such as rodents, birds, and reptiles, followed by dogs and cats [104], though other authors argue that cats and dogs are the most frequent victims [16]. Animal cruelty directed against anthropomorphized species (dogs and cats), especially in an up-close manner, is a more apt red flag of the development of extreme violence [105]. In general, boys are twice as likely to be involved in animal cruelty as girls [8,70,75]. This could be due, at least in part, to the socialization of boys which often emphasizes dominance and aggression [106]. This notion is supported by Riggs et al. [107], who found a statistically significant relationship between the perpetration of acts of animal cruelty and gender (*p* < 0.001) which suggests that men were 24% more likely to mistreat animals and perform aggressive acts.

As these studies show, we must understand that multiple factors can influence children to commit acts of animal cruelty, though we can conclude that an inappropriate child-rearing environment and previous exposure to violence will often lead the affected children to normalize acts of cruelty directed towards other living beings. A possible solution to, and prevention of, problems of animal cruelty could consist in having children participate in educational programs at school where they are taught to treat animals with respect, compassion, and kindness [108].

### 4.1. Empathy

Empathy is the aptitude to understand the feelings or emotions of other beings; that is, to comprehend what someone else feels in a given situation. Children’s awareness of animal sentience is associated with compassion and humane behaviors towards animals. Thus, considering children’s beliefs about other species in the context of animal abuse can contribute to understanding animal cruelty onset and persistence in childhood [109]. Authors such as Miralles et al. [110] mention that empathy towards animals can vary according to their taxonomic order; for example, the difference in comparing empathy that one might feel for a pet dog compared to a reptile. Despite this difference, abusing an animal of any species is a significant act that may be an indicator of a potential abuser. There is proof that the absence of empathy can play a critical role in the appearance of aggressiveness and antisocial behavior in children, adolescents, and adults [111,112]. Two types of empathy have been identified: affective and cognitive. Studies have found that people with antisocial behavior—such as bullying—and distinct levels of psychological pathologies tend to have deficits of affective empathy [72]. Animal abuse is another form of antisocial behavior and one of the main points of view for diagnosing behavioral disorders.

A possible link between a low degree of empathy and animal abuse was recently demonstrated in studies by, for example, Plant et al. [113]. Those authors studied children aged 13–17 from two cultures to analyze factors that are involved in the appearance of animal abuse. They found that 10–57% of those children had witnessed family violence and animal cruelty and had developed a negative relationship between the presence of animal abuse and affective empathy (OR = 0.19, *p* < 0.001). A similar result was obtained by Hawkins et al. [114], who conducted an exploratory study of 540 people from all six continents. They found a positive correlation between low empathy towards animals and the potential for performing acts of abuse. Although the evidence currently available is not conclusive on this relationship, there seems to be little doubt that the inability to understand the emotions that other beings feel when subjected to violence can be considered a primary red flag for detecting possible abusers.

### 4.2. Callous-Unemotional Traits

Cruelty and insensitivity refer to behaviors that demonstrate an absence of guilt or empathy in individuals who use others for their personal gain [115]. Cruelty and insensitivity are classified as callous-unemotional traits that can be understood as the antonym of empathy [116]. Individuals with these qualities are unable to recognize stress or discomfort in others [117]. In this context, Zhang et al. [118] analyzed the link between antisocial traits in 176 children aged 4–5 years. They observed a negative association between prosocial insensitivity and behavioral traits (r^2^ = −0.57, *p* < 0.01), but a positive correlation with behavioral problems such as aggression (r^2^ = 0.24–0.67, *p* < 0.05). Likewise, a study by Fanti [119] found that adolescents who presented insensitivity traits showed a high probability of manifesting behavioral problems such as impulsivity, narcissism, aggressiveness, and inattention.

Insensitivity traits are a factor of great interest in psychology because some studies sustain that a relationship exists between the presence of these antisocial behavioral traits in children and tendencies towards aggression, criminality, psychopathic behaviors [36,120], and acts of animal abuse. According to Frick and Ray [121], these are behaviors that are known to occur in the life trajectories of psychopaths. This line of research has led to the elaboration of insensitivity tests. When detected in an early stage, insensitive behaviors may make it possible to predict which individuals might commit acts of aggression against animals or other people [122].

The foregoing emphasizes that insensitivity is a particularly important trait that may generate evident signs that help determine whether a child or adolescent will be capable of performing acts of abuse towards animals that could later evolve into acts of aggression against people or crimes on a larger scale.

## 5. Warning Signs of the Potential for Domestic Violence and Animal Abuse

Scientific evidence shows an association between domestic violence and animal mistreatment. For example, children raised in home environments where violence is common tend to develop abusive and aggressive temperaments. Children from households with domestic violence who have perpetuated acts of animal abuse with their pets, exposed to interpersonal violence, and acts of animal abuse tend to normalize physical punishment in animals [123]. Animal abuse by children is a sensitive issue due to evidence that indicates that aggression towards animals during childhood could be an early indicator of the future development of violent tendencies [30,124]. According to Mead [26], animal cruelty in children is a behavior that can be diagnosed and treated early to prevent the development of violent behavior, or even homicidal tendencies, later in life. Table 2 summarizes the warning signs that have been correlated with interpersonal violence.

As mentioned above, lack of empathy is a characteristic that has been strongly associated with psychopathic behavior, but it is also symptomatic of other conditions, such as hyperactivity syndrome and related behavioral disorders [136,137]. This means that additional, specific traits must be defined to determine whether domestic violence is the root of other expressions of violence. On this topic, Rigdon and Tapia [127] studied 18 children who had participated in animal abuse. They found that they all manifested antisocial behaviors: lack of temper control, bullying, destructive tendencies, compulsive lying, and cruelty to other children and animals. MacDonald [15,138] has analyzed the topic of animal cruelty extensively. He affirms that three behaviors during childhood may be keys to predicting acts of violence against humans: (1) animal cruelty; (2) secondary enuresis (bedwetting) after the age of 5; and (3) pyromania, these factors are associated with the emotional stress caused by traumatic events that children below age 5 may experience. Recognizing them offers a potential intervention point for prompt management to promote empathy for, and respect towards, other living beings [125,138]. Research has determined that adult pyromaniacs, sexual sadists, and serial killers tend to present this triad at young ages [126].

A study of 280 people who had committed murder produced evidence that validated an association with McDonald’s triad, though it added that physical and psychological abuse by parents was also prevalent in these individuals [27]. Similarly, Walters [139] performed a multiple regression analysis on 496 sex offenders to determine whether animal cruelty and pyromania can be confirmed as markers of fearlessness and disinhibition. He found that animal cruelty is closely related to loss of fear (r = 0.20), while pyromania correlated with disinhibition (r = 0.17). Studies of this kind confirm the applicability of McDonald’s triad as a tool for detecting signs of potential abuse in children; however, studies of these psychosocial disorders must consider numerous factors to avoid reaching misleading interpretations [22].

**Figure 4:**
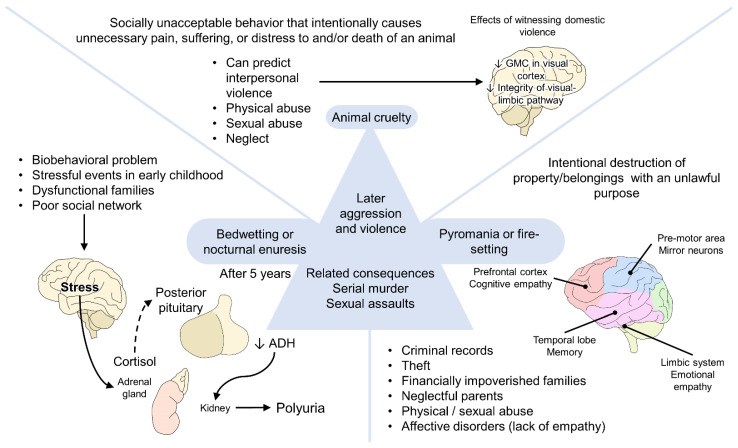
McDonald triad consists of bedwetting (nocturnal enuresis), pyromania, and animal abuse. ADH: antidiuretic hormone: GMC: gray matter concentration.

In Figure 4, the components of the McDonald triad and how they can be related are shown. As marked in previous sentences, animal cruelty is associated with an escalation of violence and could be used as an early predictor of interpersonal violence and further abusive acts [124,140]. Firstly, apart from the sociopsychological alterations than can be observed in these children, Teicher et al. [141] have found that childhood maltreatment—including verbal abuse, witnessing domestic violence, and childhood sexual abuse—can have potential effects on brain development, particularly in the structure and functionality of the grey matter (reducing its volume), the inferior longitudinal fasciculus (reducing its integrity), and the somatosensory cortex (reducing the thickness of the cortex). Likewise, young adults witnessing domestic violence were found to show an altered visual-limbic pathway and, therefore, alteration in the integration of emotional experiences [142]. The exposition to stressful or traumatic events during early childhood, such as abuse or domestic violence, is known to have a stronger association with bedwetting, where evidence also mentions the role of cortisol—secreted after stressful events—and its involvement in nocturnal polyuria [143,144]. Finally, pyromania, or fire-setting, is a characteristic that has been reported in individual victims of neglectful parenting, as well as those with affective disorders [145], where a lack of empathy and the altered limbic system circuits can influence, although the contributions of cognitive and affective empathy are still not well understood [146].

Nonetheless, some studies have also highlighted the lack of association between the so-called McDonald triad with violence. In a study by Parfitt et al. [147], the authors stated that the validity of the triad is controversial and cannot always be applied for the prompt recognition and prevention of interpersonal violence since each of the factors can occur on its own and develop antisocial behaviors and because the simultaneous presentation of the three elements is a rare event in children. Similarly, Joubert et al. [148] mention the inconsistency of distinguishing the three markers to identify potential sex offenders in a study carried out in the United States where, for example, more than half of the individuals did not experience enuresis during childhood or adolescence, and some did not show any of the three elements. Therefore, although the McDonald triad is often associated with antisocial and aggressive traits, the presentation of one or two markers is common, and they can be a result of multiple contexts and needs to be addressed in a broader perspective [149].

### Bestiality

Beirne [9] defines bestiality as an interspecies sexual assault in which the human penis is introduced into an animal’s vagina, anus, or cloaca. This act causes pain and suffering to the animal but cannot be reported because the victims—non-human animals—cannot express themselves verbally. During the act, the perpetrator has a sense of domination over the animal [150]. We need to clarify that bestiality differs from zoophilia since it does not contemplate the motivations that may lead a person to seek sexual contact with an animal [128,151]. A more appropriate definition of bestiality, therefore, could be animal sexual abuse [152]. Whichever term is used, bestiality may also be a red flag for the development of violent behaviors and a precursor to interpersonal violence in adults [133]. It has been reported that humans who perform bestiality lack empathy, are insecure, and tend to be ashamed or afraid of rejection [10].

Statistics have shown that 8% of men in the United States admit to having had some type of sexual contact with animals. Of those, 40–50% lived on rural farms [129]. A study by Hensley et al. [131] found that of 261 convicts in correctional facilities in the southern U.S., 16 said they had committed acts of bestiality as children. Henderson et al. [93] reported similar results in a sample of 180 prisoners in a different prison, where 1 in 5 admitted to having performed sexual acts with animals. The scientific literature affirms that bestiality is another warning sign that correlates with violent acts towards adults and children [132]. Fleming et al.’s [130] study of 24 young people who had performed acts of bestiality found that 23 had participated in sexual assaults against humans. Edwards [153] also reported this association. His analysis of sexual predators arrested in 2003–2017 observed that 2.6% had antecedents of participating in bestiality and that those individuals were significantly more prone to have been victims of child sexual violence (*p* < 0.005), to have engaged in animal cruelty (*p* < 0.0001), and to have committed child sexual abuse (*p* < 0.005).

A psychological evaluation of an 18-year-old male with antecedents of child sexual abuse found an absence of cognitive dysfunction and average intelligence but active psychopathology, but other tests revealed that he was emotionally and sexually immature, suffered from attachment disorders and a lack of empathy, and had little self-discipline and lower than normal sensitivity to criticism. This male had not shown any feeling of guilt or remorse associated with the death of a calf and admitted to the heterosexual practice which listed the case in the category of bestiality [154]. Evidence of this kind shows a clear connection between animal cruelty and interpersonal violence, though no link between interpersonal violence and bestiality has yet been established [155]. Bestiality may be a prelude to later sexual offenses, but the evidence available today does not show it to be a conclusive factor (though it often includes elements of MacDonald’s triad). For now, it is mentioned as a potential triggering factor for future violence.

## 6. Animal Cruelty and Animal Protection Laws

Since the 17th century, animal cruelty has been the justification for enacting laws to foster animal welfare. In the Middle Ages, Saint Francis of Assisi preached those animals are our brothers and sisters. In 1635 in Ireland, in 1641 in Massachusetts, and in 1659 in the United Kingdom, animal protection laws were passed due to concerns over slaughtering processes. However, it was not until 1822 that the first law enacted to prevent violent or cruel acts and practices towards livestock was ratified [156]. It did not, however, mention the term “animal cruelty” for it still considered animals to be property, not sentient beings. Gradually, a corpus of law designed to regulate the treatment of animals and impose penalties for these types of crimes was established [128]. One of the pioneering legislators in the battle to prevent animal cruelty was Henry Bergh, who founded and chaired the American Society for the Prevention of Animal Cruelty (ASPCA). In 1867, the ASPCA incorporated the term “animal cruelty” into the laws of New York to describe offenses against animals that deserved punishment. This served as an example for several states of the American Union, such as Massachusetts, Pennsylvania, Illinois, and New Hampshire, all of which proceeded to enact laws against animal cruelty [157].

Currently, the Society for the Prevention of Animal Cruelty has locations worldwide, with offices in Africa, Asia, India, Europe, North America, and Oceania. Particularly North America, Europe, Australia, and New Zealand are the regions with a high degree of animal legislation to protect companions, farm animals, and wildlife, and enforce its application throughout the countries [158]. Regardless, some gaps have been recognized. For example, in Australia, animal welfare is not included in the national constitution; every state is responsible for establishing legislation in their territory, where the lack of uniform federal legislation regarding animal protection causes shortcomings, such as the lack of definition of what is considered an animal and the penalties to reinforce the law [159]. The inconsistency in the use of particular terms such as “animal”, “suffering”, or “harm” is also seen in European countries, where the application of the law depends on the interpretation of the lawyer when there are unclear terminologies in the legislation [160]. Although the European Union recognizes animals as sentient beings that can feel pain and suffer, this is only legally appointed in the civil codes of France, Austria, Germany, Switzerland, and Catalonia [161].

In the case of Asia, countries such as China have changed their attitude towards animals, mainly companion animals, considering them from a humanistic and moralistic point of view instead of as valuable resources thanks to animal welfare courses. However, political or cultural aspects are the main reasons why rigorous animal laws are difficult to implement nationally [162]. The differences between countries depend on the sociopolitical, religious, economic, and cultural aspects [158]. In regions where Islamicists and Muslims are guided by religious beliefs, dogs are perceived differently. While dogs can be considered pets and eating their meat is prohibited, they can also be recognized as animals used exclusively for guarding and hunting, while cats, fishes, and birds are broadly accepted as companion animals [163].

In Latin America, animal protection programs and laws are minimal, although interest in preserving animal welfare has been growing. Brazil, Peru, Costa Rica, Colombia, Uruguay, and Mexico have laws, a draft of a law, or official norms that protect companion animals from cruelty acts. In Mexico, the Animal Protection Law aims to guarantee the well-being of companion animals, preventing cruelty and providing respectful treatment of animals [164]. However, although the commitment and desire of these countries to reinforce animal welfare laws, corruption, economic situation, and cultural variables determine the success of the legislation [158].

To cover these deficiencies, animal protection laws were modified throughout the 19th century as additional acts came to be considered animal abuse or cruelty; for example, not providing food, water, shelter, or veterinary services to companion animals, mutilations, bestiality, beatings, malicious killing, and any situation that causes pain or suffering. In many cases, fines and sanctions were established to punish these crimes [134]. Much remains to be done, however, to improve animal protection laws because many do not define the term animal, have confusing wording, or leave gaps. Finally, the punishments and fines imposed tend to be insufficient or difficult to apply [165]. Additionally, because these laws are written by humans, they do not necessarily favor animals as they should [166]. One way to approach this problem is to encourage laws with veterinary guidance. As professionals on animal health, veterinarians must be part of the authorities in charge of constructing animal cruelty laws so that it is possible to address the problem from a perspective based on medical knowledge and animal welfare science [167]. With this objective, courses focusing on animals’ legal rights or animal law must be promoted in school curriculums [168]. Moreover, measures to guarantee the application of laws and that the punishment, charges, or penalties for animal abusers are also being covered are needed [169].

In the connection between domestic violence and animal abuse, although domestic violence laws have expanded in recent decades, the law in most regions ignores the participation and vulnerability of animals in a violent environment. Traditionally, animals have been barred from most domestic violence shelters, making it difficult for victims to leave violent homes [170,171]. In the same way, some efforts have been made in order to understand the socioeconomic factors related to animal abuse in the family environment, and animals are excluded from social programs [172]. Despite this, some programs have been developed to house victimized humans and animals. The legislation of a few states of the United States incorporates animal abuse into domestic violence provisions and protective orders [63,173].

## 7. One Health, One Welfare Approach

Research on the connection between animal cruelty and other forms of violence has led to significant policy changes in the USA. These include upgrading certain acts of animal abuse to felonies, allowing pets to be included on domestic violence protection orders, identifying animal cruelty as an element of domestic violence, mandating or allowing cross reporting by social services professionals and animal service agencies, referencing animal cruelty in court decisions regarding parental rights, enhancing penalties for exposing children to animal cruelty, and adding animal cruelty to the Federal Bureau of Investigation’s (FBI) and National Incident-Based Reporting System (NIBRS) [42,46]. However, in most regions, there is still a lack of attention to animal abuse within family violence because of the assignment of functions [42]. Human protective services are primarily a function of the government social and health institutions, while animal welfare services belong to the private human societies or public environment.

Diverse studies point out the importance of maintaining an interdisciplinary and multidisciplinary approach in the timely recognition of domestic violence, from a public health perspective [174,175,176,177]. Such is the case of the professional practice of the dentist, since, on many occasions, it is in the mouth where signs of physical violence can be detected [178,179], or of veterinarians when detecting animal abuse [70,166,167], which has been closely related to the tendency to develop criminal acts later [68]. Training and awareness are essential for health professionals, their position to face intrafamily violence and hence the importance of carrying out more research and programs for this purpose, as well as systematic and effective actions with the purpose of humanizing and taking care of the health of mistreated people [152].

Early life humane education and animal-assisted programs are considered an option to promote childhood awareness of the importance of empathy, kindness, and love towards animals and people [180]. The main goal of these education programs is to build a sense of empathy and teach compassion within and outside families [181]. Humane educators with a greater tendency to show kindness and welfare-orientated attitudes constitute a positive approach for schools to instill these traits in young people [182]. Similarly, animal-assisted psychotherapy with species such as equines has shown to help children involved in traumatic experiences including domestic violence [183]. When children learn to take care of animals and treat them kindly, they tend to do the same with conspecifics and will show respect to every sentient being, which may help in reducing animal and human violence [184].

As the National Institute of Health states, the major recommendation is to prevent children from developing callous-unemotional behaviors [181]. However, to date, the participation of adolescents and adults in animal-based programs is encouraged, particularly in prisons, where animals such as rescue dogs are a form of treatment and rehabilitation, serving to reduce the rate of violence and suicide attempts inside these institutions [185]. Additionally, education with animals has physical (e.g., weight loss), psychological (e.g., reduces stress), social (e.g., social intelligence), behavioral, and environmental benefits for the inmate, their family, animals, and society [186]. For example, the jail-based course Parenting, Prison, and Pups has shown to be an effective parenting therapy for mothers housed in jails to correct and develop a nourishing relationship with their children [187]. As we have seen, if children grow up in healthy and positive environments where they are taught the importance of human and animal care, the likelihood of developing negative behaviors is reduced.

Likewise, fostering empathy is linked to people’s prosocial functioning [188], including volunteering, helping, and reacting sensitively to animals, humans, and the environment [180,189,190]. A one health, one welfare approach is a set of multidisciplinary actions and systems that promote human health and well-being, animal welfare, human–animal relationships, and environmental protection [191,192,193,194,195]. Therefore, it is essential to form a network of veterinarians, human healthcare professionals, local social services, legal experts, and academics from related fields to enhance the awareness, identification, and intervention plans for the cycle of animal abuse and domestic violence [184,189,190,192,193,194,195].

## 8. Conclusions

Animal cruelty is intimately associated with domestic violence and represents another form of abuse that may accompany harmful treatment of children or romantic partners and forms of psychological abuse and intimidation. Likewise, animal abuse and domestic violence are considered predecessors of criminal and violent traits in humans that require prompt attention. The indicators described by MacDonald have demonstrated their validity for the early identification of potentially violent individuals. The correlation among pyromania, secondary enuresis, and animal cruelty is clear, but we can now add a lack of empathy and the tendency to escalate to even crueler acts, such as child abuse or even murder. The factors that influence manifestations of violent behavior towards humans and animals are often linked to violent domestic environments. When children are raised in environments where aggression and abuse are part of the daily routine, it can alter the way in which they socialize with others. Moreover, indicators such as insensitivity and poor social skills must be considered as elements of any strategic plan designed to prevent or correct animal abuse in children.

One of the approaches recommended to prevent animal abuse and cruelty in children and teenagers is early childhood education. Teaching children to be more humane, compassionate, and respectful towards living beings can help reduce all types of violence. However, there is much room for improvement in promoting the well-being of humans and animals that have been, or are currently, victims of abuse or cruelty. Teaching programs and laws are needed to encourage and educate society, but veterinarians must also take on an active role in the process of identifying and preventing animal abuse.

## Figures and Tables

**Table 1:** Percentage of domestic violence victims who have witnessed animal abuse in five regions.

Country or City	Percentage	Reference
Australia	52.9%	Volant et al. [2]
New York	53%	Carlisle-Frank et al. [66]
Utah	54%	Ascione et al. [16]
Ireland	57%	Allen et al. [29]
Texas	36%	Faver and Strand [21]

**Table 2:** Studies investigating warning signs to identify and prevent interpersonal (domestic or community violence).

Warning Sign	References
MacDonald triad (bedwetting, animal cruelty, setting fires	MacDonald [15]; Felthous and Bernard [125]; Ressler et al. [126]
Animal cruelty	Pinel [25]; MacDonald [15]; Mead [26]; Rigdon and Tapia [127]; Baldry [78]; Ascione [16,75]; Gallagher et al. [7]; Febres et al. [17]; McDonald et al. [18]; Monsalve et al. [19]; Newberry [20]; McDonald et al. [123]
Bullying	Rigdon and Tapia [127]; Baldry [78]; Walters [80]
Destructive behavior	Rigdon and Tapia [127]
Poor temper control	Rigdon and Tapia [127]; Holoyda and Newman [128]
Bestiality	Kinsey et al. [129]; Beirne [9]; Fleming et al. [130]; Hensley et al. [131]; Abel [132]; Hensley et al. [133]; Henderson et al. [93]; Holoyda and Newman [134]
Compulsive lying	Rigdon and Tapia [127]
Lack of empathy	Jolliffe and Farrington [111,112]; Hartman et al. [30]
Cruelty, insensitivity	Barry et al. [120]; Frick et al. [36]; Muñoz et al. [72]; Hartman et al. [30]
Aggressive or violent tendencies	Barry et al. [120]; Frick et al. [36]; Gupta [135]

## Data Availability

Not applicable.
